# Constructing a novel mitochondrial-related gene signature for predicting survival and evaluating the tumor immune microenvironment in clear cell renal cell carcinoma

**DOI:** 10.3389/fgene.2025.1543593

**Published:** 2025-09-22

**Authors:** Jiaxuan Qin, Lijian Zhang, Bowen Chen

**Affiliations:** Department of Urology Surgery, the First Affiliated Hospital of Xiamen University; School of Medicine, Xiamen University; Center of Diagnosis and Treatment of Urinary System Diseases, the First Affiliated Hospital of Xiamen University; the Key Laboratory of Urinary Tract Tumors and Calculi of Xiamen City, the First Affiliated Hospital of Xiamen University, Xiamen, Fujian, China

**Keywords:** mitochondrial-related gene, survival, tumor immune microenvironment, clear cell renal cell carcinoma, bioinformatics

## Abstract

**Background:**

Mitochondria play an important role in tumors. Cellular energy supply, signaling, metabolism, autophagy, aging, and tumorigenesis are all associated with mitochondria. However, we lack a reliable prognostic model using mitochondrial-related genes in clear cell renal cell carcinoma (ccRCC).

**Methods:**

A systematic analysis of available TCGA databases and related studies was conducted using the R language and online analysis tools to evaluate the prognostic value of mitochondrial-related genes and the tumor microenvironment in ccRCC.

**Results:**

We constructed a novel mitochondrial-related gene signature for predicting survival and evaluating the tumor immune microenvironment in ccRCC. The mitochondrial-related gene signature included MICALL2, FKBP10, and ACADSB. According to the risk score of the risk model, ccRCC patients were divided into high- or low-risk groups. The ccRCC high-risk group with a high-risk score is related to poor prognosis and poor efficacy from immune checkpoint inhibitors (ICIs).

**Conclusion:**

Our mitochondrial-related gene signature, as a risk model, could be a reliable ccRCC prognostic biomarker and could predict the response to immunotherapy. The risk score was correlated with the tumor microenvironment and immune cell infiltration.

## 1 Introduction

Mitochondria play an important role in tumors (Zong et al., 2016). Cellular energy supply, signaling, metabolism, autophagy, aging, and tumorigenesis are all associated with the mitochondria. The tumor microenvironment affects immune cell evasion or inhibition and drug resistance in malignancies. There are interactions between the tumor microenvironment and mitochondrial function in tumor cells (Yang et al., 2023). Different pathological types of renal tumors have their own characteristics of mitochondrial morphology (Nikolic et al., 2023). Clear cell renal cell carcinoma (ccRCC) exhibits a substantial reduction in mitochondrial mass, paralleled by a reduction in both mtDNA copy number and RNA transcription. Some ccRCC samples display a significant elevation in acyl-carnitines, pointing to a ccRCC-specific disruption of mitochondrially localized beta-oxidation, in line with the reduction in mitochondrial mass (DiNatale et al., 2020). The hypoxic background promoted by tyrosine-kinase inhibitors (TKIs) might be altering the tumor microenvironment in a way that facilitates tumor recognition by immune cells, ultimately leading to improved response in renal cell carcinoma (DiNatale et al., 2020). Mitochondrially targeted anticancer drugs that disrupt the energy-producing systems of cancer are emerging as new potential therapeutics in renal cell carcinoma (Bielcikova et al., 2023). However, we lack a reliable prognostic model in ccRCC that uses mitochondrial-related genes.

## 2 Materials and methods

### 2.1 Flow chart and main steps

A flow chart of the work is shown in Supplementary Figure S1. The main steps are to identify differentially expressed genes (DEGs) between TCGA ccRCC normal and tumor samples and then intersect the list of DEGs with mitochondrial-related genes (MRGs) to identify common DEGs. A few genes were further selected by Lasso regression analysis in the common DEGs. Then, a prognostic mitochondrial-related gene signature was constructed and validated based on the selected genes. The TCGA ccRCC samples were divided into low- and high-risk groups based on the prognostic mitochondrial-related gene signature. Gene Ontology (GO) and Kyoto Encyclopedia of Genes and Genomes (KEGG) pathway analyses were conducted. Finally, the tumor microenvironment analysis and immunotherapy responses of the low- and high-risk groups were predicted.

### 2.2 Data collection

RNA-seq data and clinical information about the ccRCC TCGA training cohort were downloaded from the GDC data portal (https://portal.gdc.cancer.gov/), and 521 ccRCC tumor samples with enough clinical information were used in the survival-related analysis and the tumor immune microenvironment-related analysis. The RNA array assay data and clinical information of the 101 ccRCC tumor samples in the E-MTAB-1980 validation cohort were downloaded from the ArrayExpress database (https://www.ebi.ac.uk/biostudies/arrayexpress/) of EMBL-EBI (European Bioinformatics Institute of the European Molecular Biology Laboratory). The list of 2030 MRGs was identified in a published study (Chang et al., 2023), and the data were collected from the MitoCarta 3.0 database and the Gene Set Enrichment Analyses (GSEA). Protein level expression data, including figures and immunohistochemical (IHC) images, were obtained from CPTAC through UALCAN (https://ualcan.path.uab.edu/) and HPA (https://www.proteinatlas.org/).

### 2.3 Identification of differentially expressed genes

We used the “DESeq2” package of R to identify DEGs between normal and tumor samples, or between high- and low-risk tumor groups from the TCGA training cohort. Only genes that were expressed >0 in all samples were retained. The criteria for defining DEGs were |Log_2_ fold change|>1 and adjusted P < 0.05. For visualizing the DEGs, we used the “ggplot2” package of R to create the volcano plot, which used |Log_2_ fold change|>1 and adjusted P < 0.05 as boundaries. The list of 2,955 DEGs was also obtained from GEPIA2 (http://gepia2.cancer-pku.cn/) (Tang et al., 2017) by using the ANOVA method with |Log_2_ fold change|>1 and q-value<0.01 for further screening of DEGs. A Venn plot was created using the EVenn online tool (Chen et al., 2021) to display the DEGs and mitochondrial-related genes (MRGs) common to both DEG groups.

### 2.4 Construction and validation of prognostic mitochondrial-related gene signature

The common DEGs were further screened by Lasso regression analysis using the “glmnet” and “survival” packages of R and multivariable Cox regression analysis (the coxph function in the “survival” package of R). The coefficient index was achieved by using the cph function in the “rms” package of R. The specific multivariable Cox regression cph statement is as follows: “cph (Surv (OS.time, OS)∼gene 1+gene 2+…+gene n, data name).” Risk score = ∑gene n × βn. The “gene” represents the log_2_ (TPM+1) value or other normalized data value of genes selected into the mitochondrial-related risk score signature. The “β” represents the coefficient index value. The “n” represents the serial number. The samples were divided into low- and high-risk groups based on the risk score (median cut-off value). To analyze the survival condition for the prognosis signature, a Kaplan–Meier (K-M) survival curve was constructed by using the “survival” and “survminer” packages of R. The predictive performance was presented by the ROC curve. The prognostic mitochondrial-related risk score signature was further validated in the E-MTAB-1980 cohort, and the data of this cohort were normalized through the robust multi-array average (RMA) algorithm.

### 2.5 GO and KEGG pathway analyses

DEGs between the high- and low-risk groups were input into the DAVID website (https://david.ncifcrf.gov/) for Gene Ontology (GO) and Kyoto Encyclopedia of Genes and Genomes (KEGG) pathway analyses. Results were visualized by using the “ggplot2” and “GOplot” packages of R.

### 2.6 Tumor microenvironment analysis and immunotherapy response prediction

The stromal score, immune score, and ESTIMATE score were calculated by using the “estimate” package of R. Tumor purity = cos (0.6049872018 + 0.0001467884×ESTIMATE score) (Yoshihara et al., 2013). A box plot was created by using the “ggpubr” package of R. A correlation scatter plot was achieved by using the “ggpubr” and “ggExtra” packages of R.

The abundance of 22 tumor-infiltrating immune cells (TIICs) was calculated by using the CIBERSORT algorithm (Newman et al., 2015) in R (https://cibersortx.stanford.edu/), and the results were visualized as a box plot by using the “ggpubr” package of R. The Wilcoxon test was used for assessing between-group differences.

The list of 79 immune checkpoint genes (ICGs) was obtained from a published study (Hu et al., 2021). The statistically significant results of 39 genes were visualized as a box plot by using the “ggpubr” package of R. A t-test was used to determine statistically significant correlations between groups.

The immunotherapy response prediction was analyzed by the tumor immune dysfunction and exclusion (TIDE) score (http://tide.dfci.harvard.edu/login/). A violin plot for TIDE was achieved by using the “ggpubr” package of R. A bar chart to visualize the immunotherapy response prediction results was created by using the “ggplot2,” “ggthemes,” and “ggprism” packages of R.

### 2.7 Statistics analyses

R (version 4.3.2) software was used for all statistical analyses. Some test methods are annotated in the figures and tables. The t-test was used to analyze the expression and distribution of TIDE score, exclusion score, dysfunction score, and microsatellite instability (MSI) score in different groups. Pearson’s chi-squared test was used to evaluate the immunotherapy response difference in TIDE prediction. The correlation was evaluated using the Spearman method. P < 0.05 was defined as statistically significant. A count value was used in the “DESeq2” package analysis. The TPM value was used in the CIBERSORT algorithm. Data were normalized according to the requirements in the TIDE algorithm. In other analyses, the log_2_ (TPM+1) value was used.

## 3 Results

### 3.1 Identification of DEGs related to mitochondria

**FIGURE 1:**
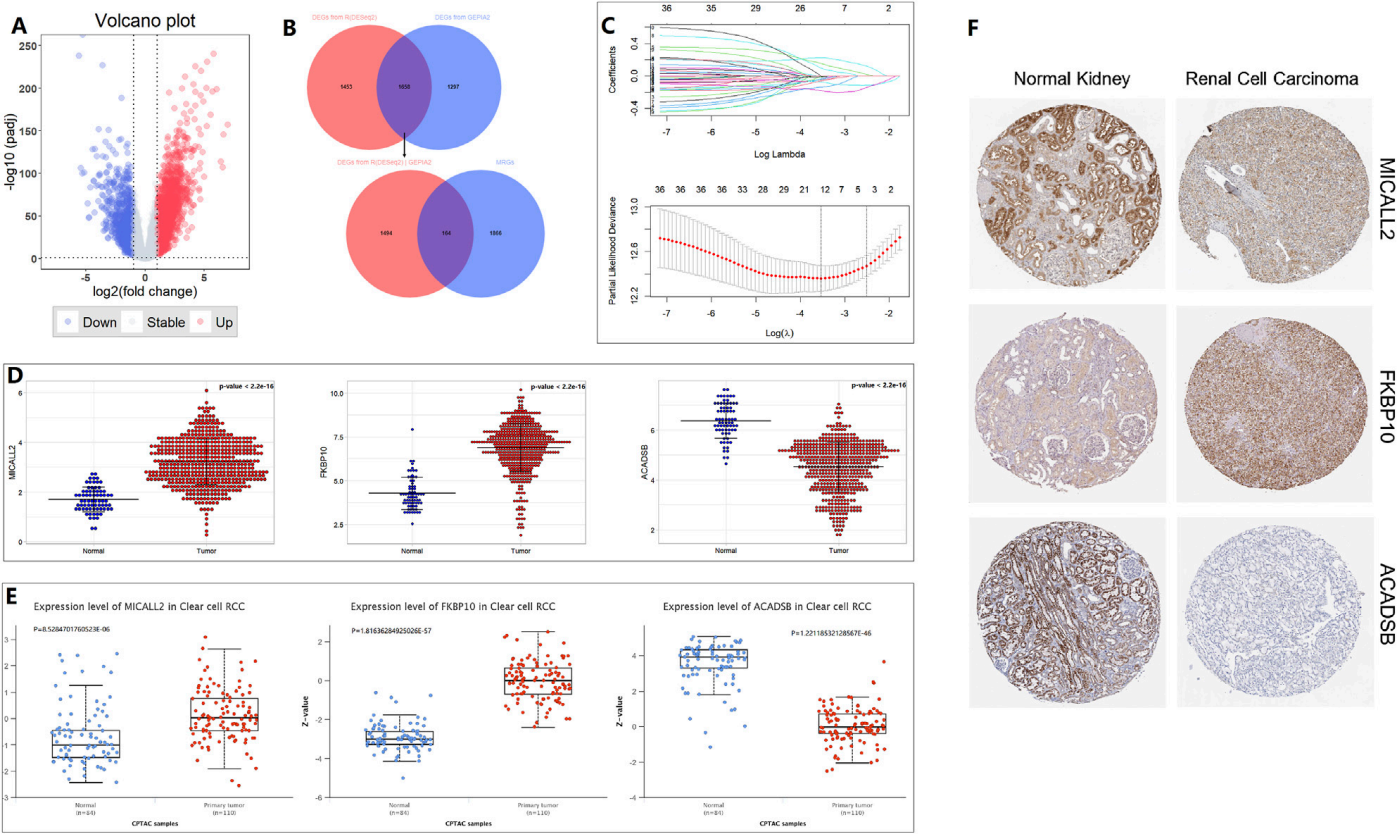
Mitochondrial-related gene selection and expression level. **(A)** Volcano plot. **(B)** Venn plot. **(C)** Lasso regression analysis. **(D)** RNA-level expression. **(E)** Protein-level expression from CPTAC. **(F)** Protein-level expression in IHC from HPA.

**FIGURE 2:**
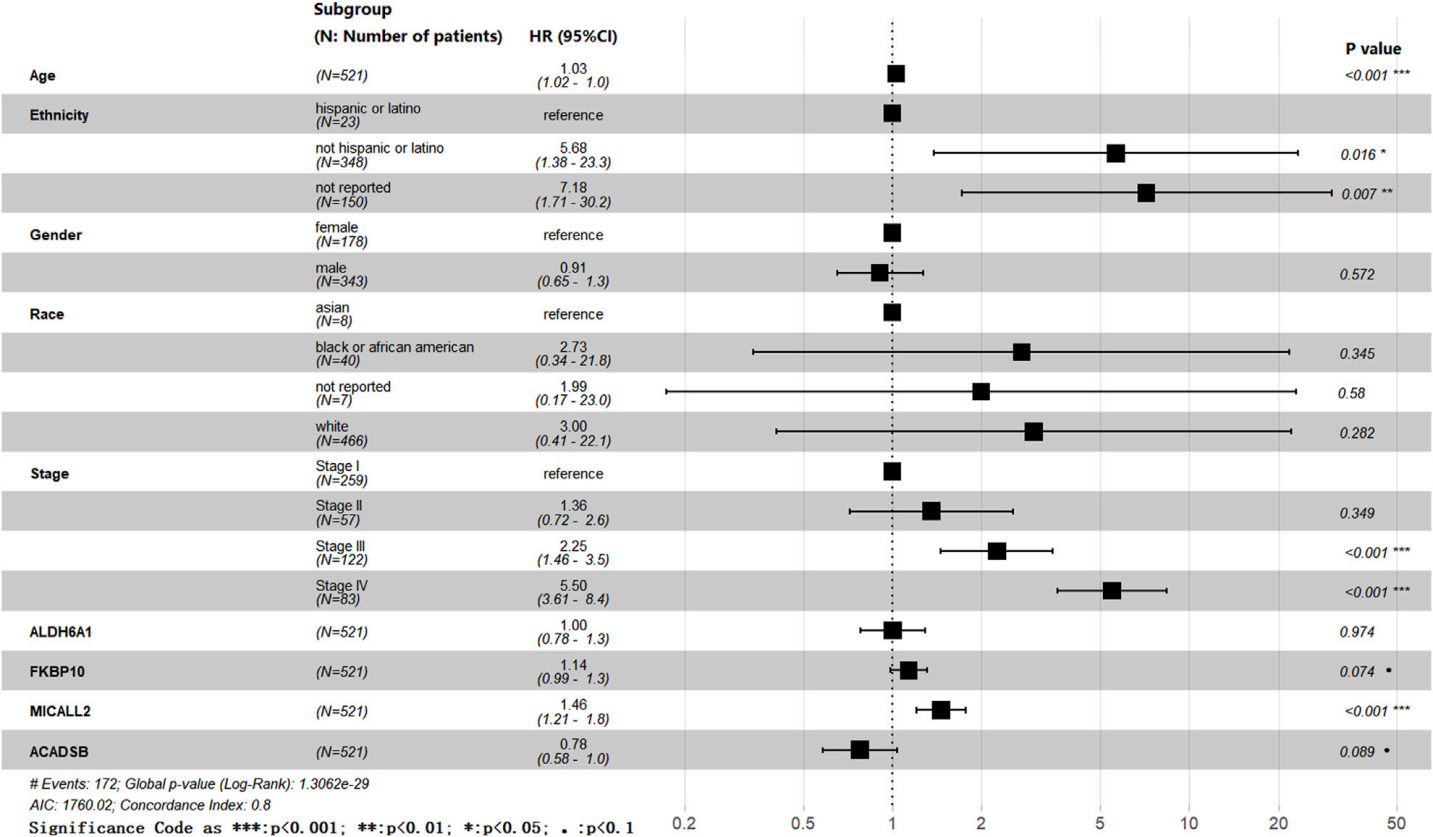
Forest plot.

By using the “DESeq2” package of R in the TCGA training cohort, 3,111 DEGs (986 down; 2,125 up) were obtained from 14,449 genes expressed in the normal and tumor groups. The DEGs are visualized by a volcano plot (Figure 1A). A total of 1,658 common DEGs between the 3,111 DEGs from the “DESeq2” package analysis and the 2,955 DEGs from the ANOVA method analysis in GEPIA2 are shown in the Venn plot. We found 164 common DEGs between the 1,658 DEGs and the 2030 MRGs (Figure 1B). In the 164 common DEGs, we ultimately selected 41 genes with |Log_2_ fold change|>2 for Lasso regression analysis and multivariable Cox regression analysis. Through Lasso regression analysis (Figure 1C), four genes were filtered out: MICALL2, FKBP10, ACADSB, and ALDH6A1. Through multivariable Cox regression analysis (coxph function in the “survival” packages of R, Figure 2), three mitochondrial-related DEGs, including MICALL2, FKBP10, and ACADSB, were finally selected to build a ccRCC prognostic model. The RNA level expression (Figure 1D) and protein level expression (Figures 1E,F) of three DEGs between normal samples and tumor samples are shown in Figure 1. In renal cell carcinoma (unknown pathological subtype) IHC images from HPA (Figure 1F), eight with MICALL2 medium staining and four with MICALL2 low staining; six with FKBP10 medium staining and six without FKBP10 staining; nine without ACADSB staining, one with ACADSB low staining, one with ACADSB medium staining, and one with ACADSB high staining. The information about the three genes is shown in Table 1.

**TABLE 1:** The information on three prognosis-related genes.

Gene symbol	Gene ID	Full name	Location	Function of the encoded protein
MICALL2	79,778	MICAL Like 2	Cytosol, endosome, nucleus, cytoskeleton, and plasma membrane	Enables filamin binding activity. Involved in the positive regulation of protein targeting mitochondria. Predicted to be located in several cellular components, including the bicellular tight junctions, neuron projections, and recycling endosomes. Predicted to colocalize with stress fiber.
FKBP10	60,681	FKBP prolyl isomerase 10	Endoplasmic reticulum and mitochondria	Belongs to the FKBP-type peptidyl-prolyl cis/trans isomerase (PPIase) family. This protein localizes to the endoplasmic reticulum and acts as a molecular chaperone. Alternatively spliced variants encoding different isoforms have been reported.
ACADSB	36	Acyl-CoA dehydrogenase short/branched-chain	Mitochondria	Short/branched-chain acyl-CoA dehydrogenase (ACADSB) is a member of the acyl-CoA dehydrogenase family of enzymes that catalyze the dehydrogenation of acyl-CoA derivatives in the metabolism of fatty acids or branched-chain amino acids. Substrate specificity is the primary characteristic used to define members of this gene family. The ACADSB gene product has the greatest activity towards the short branched-chain acyl-CoA derivative, (S)-2-methylbutyryl-CoA, but also reacts significantly with other 2-methyl branched-chain substrates and with short straight-chain acyl-CoAs. The cDNA encodes a mitochondrial precursor protein that is cleaved upon mitochondrial import and predicted to yield a mature peptide of approximately 43.7 KDa.

The effects of age, ethnicity, gender, race, stage, ALDH6A1, FKBP10, MICALL2, and ACADSB were all involved in the forest plot (Figure 2), and we obtained a list of more variable coefficient index values from this analysis. Age, ethnicity, stage, and MICALL2 showed statistically significant differences. The P-values of FKBP10 and ACADSB were less than 0.1. Meanwhile, we also obtained a list of multivariable coefficient index values by using the cph function in the “rms” package of R, and only the effects of MICALL2, FKBP10, and ACADSB were involved in this analysis.

### 3.2 Construction and validation of prognostic mitochondrial-related gene signature

**FIGURE 3:**
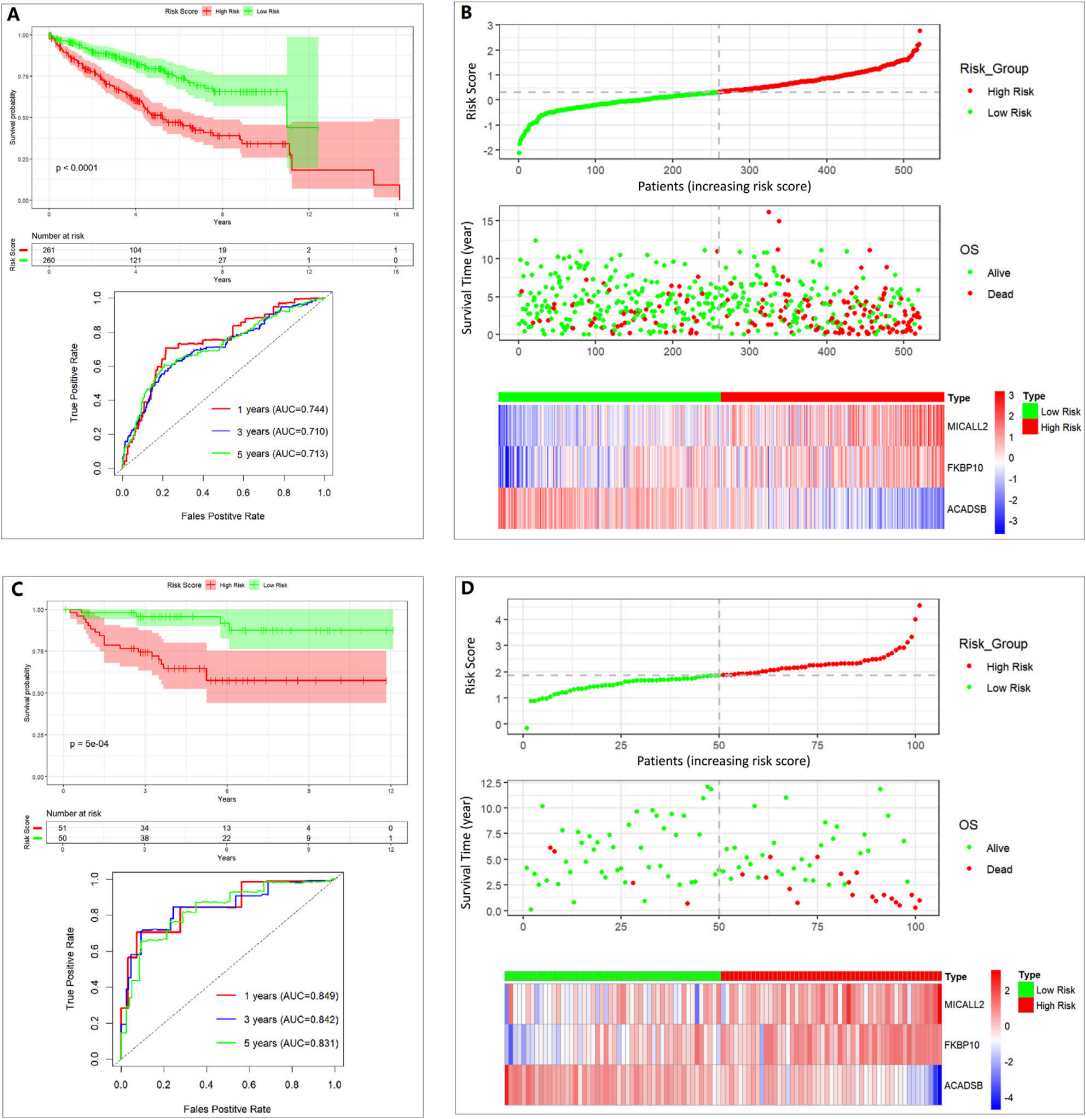
Assessing the performance of the prognostic risk model in the training and validation cohort. **(A)** K-M curve and ROC curve in the TCGA 521 ccRCC cohort. **(B)** Distribution of risk score, survival status, and expression of three genes in the TCGA 521 ccRCC cohort. **(C)** K-M curve and ROC curve in the E-MTAB-1980 101 ccRCC cohort. **(D)** Distribution of risk score, survival status, and expression of three genes in the E-MTAB-1980 101 ccRCC cohort.

The more variable coefficient index value and the multivariable coefficient index value were validated separately through a Kaplan–Meier (K-M) survival curve and the ROC curve. Risk score (more variable coefficient index value, Supplementary Figure S2) = 0.379045×MICALL2 + 0.127374×FKBP10 − 0.249544×ACADSB. Risk score (multivariable coefficient index value, Figures 3A,B) = 0.3089×MICALL2 + 0.1995×FKBP10 − 0.4429×ACADSB. The samples were divided into low- and high-risk groups based on the risk score (median cut-off value). In Supplementary Figure S2, we show the intersection between the K–M survival curve and the lower AUC values of the ROC curve. Finally, a multivariable coefficient index value was selected. Then, the risk score for each ccRCC patient in both the training and the validation cohorts was computed based on the following formula:

Risk score=0.3089×MICALL2+0.1995×FKBP10 ‐ 0.4429×ACADSB.

The stability of the prognostic risk model was further validated in the E-MTAB-1980 cohort, and similar results are shown in the K–M survival curve and the ROC curve (Figures 3C,D).

Clinical characteristics of the low- and high-risk groups in the TCGA 521 ccRCC cohort are shown in Table 2, which was created using the “gtsummary” package of R with univariate Cox regression analysis. The high-risk group had a higher proportion of men, T3+T4 stage, M1 stage, stage III + IV, and dead status.

**TABLE 2:** Clinical characteristics between low- and high-risk groups.

Characteristic	High risk, N = 261^a^	Low risk, N = 260^a^	P-value^b^
Age			0.69
Mean (SD)	60 (12)	61 (12)	
Median (IQR)	60 (53, 69)	61 (51, 71)	
Range	26, 88	29, 88	
Gender			**0.009** ^c^
Female	75 (29%)	103 (40%)	
Male	186 (71%)	157 (60%)	
Race			0.96
Asian	4 (1.5%)	4 (1.5%)	
Black or African American	21 (8.0%)	19 (7.3%)	
White	233 (89%)	233 (90%)	
Missing	3 (1.1%)	4 (1.5%)	
Ethnicity			0.41
Hispanic or Latino	10 (3.8%)	13 (5.0%)	
Not Hispanic or Latino	182 (70%)	166 (64%)	
Missing	69 (26%)	81 (31%)	
T Stage			**<0.001**
T1+T2	135 (52%)	199 (77%)	
T3+T4	126 (48%)	61 (23%)	
N Stage			0.15
N0	122 (47%)	117 (45%)	
N1	11 (4.2%)	4 (1.5%)	
NX	128 (49%)	139 (53%)	
M Stage			**<0.001**
M0	190 (73%)	237 (91%)	
M1	58 (22%)	20 (7.7%)	
MX	13 (5.0%)	3 (1.2%)	
Tumor stage			**<0.001**
Stage I + II	122 (47%)	194 (75%)	
Stage III + IV	139 (53%)	66 (25%)	
Survival status			**<0.001**
Alive	140 (54%)	209 (80%)	
Dead	121 (46%)	51 (20%)	

^*a*^ n (%).

^*b*^ Wilcoxon rank sum test; Pearson’s chi-squared test; Fisher’s exact test.

^*c*^ Bold indicates P-value ≤0.05 was considered statistically significant.

Multivariate Cox regressions of various prognostic parameters in the TCGA 521 ccRCC cohort are shown in Table 3. Through multivariable Cox regression analysis (coxph function in “survival” packages of R) and the “gtsummary” package of R, the effects of age, gender, race, ethnicity, detailed stage subgroups, and risk group were examined. Stage IV and high-risk groups are related to an unfavorable prognosis. Non-Hispanic or non-Latino ethnicity and high age might be related to unfavorable prognosis; however, the left 95% confidence interval (CI) of age is near 1, and the P-value of race is not statistically significant.

**TABLE 3:** Multivariate Cox regression of various prognostic parameters.

Characteristic	HR^a^	95% CI^a^	P-value
Age	1.02	(1.00–1.04)	**0.016** ^b^
Gender			
Female	1		
Male	0.86	(0.58–1.28)	0.5
Race			
Asian	1		
Black or African American	0.79	(0.09–6.79)	0.8
White	0.75	(0.10–5.54)	0.8
Ethnicity			
Hispanic or Latino	1		
Not Hispanic or Latino	5.18	(1.24–21.7)	**0.024**
T stage			
T1	1		
T2	0.92	(0.21–3.99)	>0.9
T3	1.06	(0.31–3.68)	>0.9
T4	1.04	(0.28–3.90)	>0.9
N stage			
N0	1		
N1	1.89	(0.74–4.83)	0.2
NX	0.84	(0.57–1.23)	0.4
M stage			
M0	1		
M1	0.46	(0.09–2.39)	0.4
MX	0.49	(0.08–2.85)	0.4
Tumor stage			
Stage I	1		
Stage II	1.26	(0.24–6.65)	0.8
Stage III	2.09	(0.56–7.86)	0.3
Stage IV	10.4	(1.53–70.8)	**0.017**
Risk group			
High risk	1		
Low risk	0.5	(0.32–0.76)	**0.001**

^*a*^ HR, Hazard ratio; CI, Confidence interval.

^*b*^ Bold indicates P-value ≤0.05 was considered statistically significant.

### 3.3 GO and KEGG pathway analyses

**FIGURE 4:**
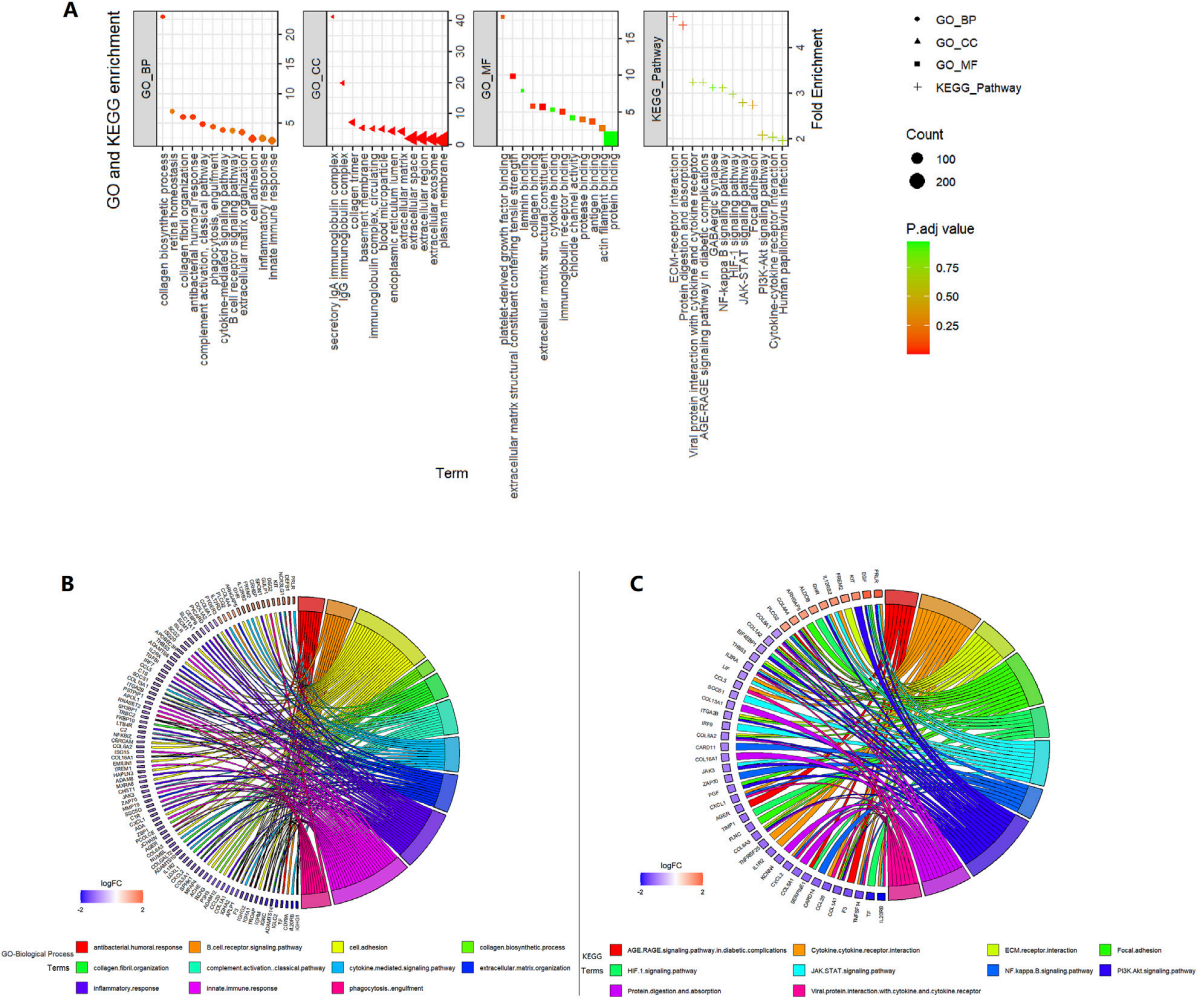
Enrichment analysis in the high- vs. low-risk group in the TCGA 521 ccRCC cohort. **(A)** GO and KEGG enrichment. **(B)** GO-biological process circle. **(C)** KEGG circle.

A total of 484 DEGs between the high- and low-risk groups were selected in the TCGA 521 ccRCC cohort and were input into the DAVID website. GO and KEGG pathway analyses results are shown in Figure 4. ECM–receptor interaction and focal adhesion pathway, which are involved in tumor invasion and tumor microenvironment, were enriched in KEGG analysis. JAK-STAT, PI3K-Akt, NF-kappa B, and HIF-1 signaling pathways were also enriched in KEGG analysis. Some extracellular-related functions, some immune-related functions, and cytokine-mediated signaling pathways were enriched in the GO analysis.

### 3.4 Tumor microenvironment analysis and immunotherapy response prediction

**FIGURE 5:**
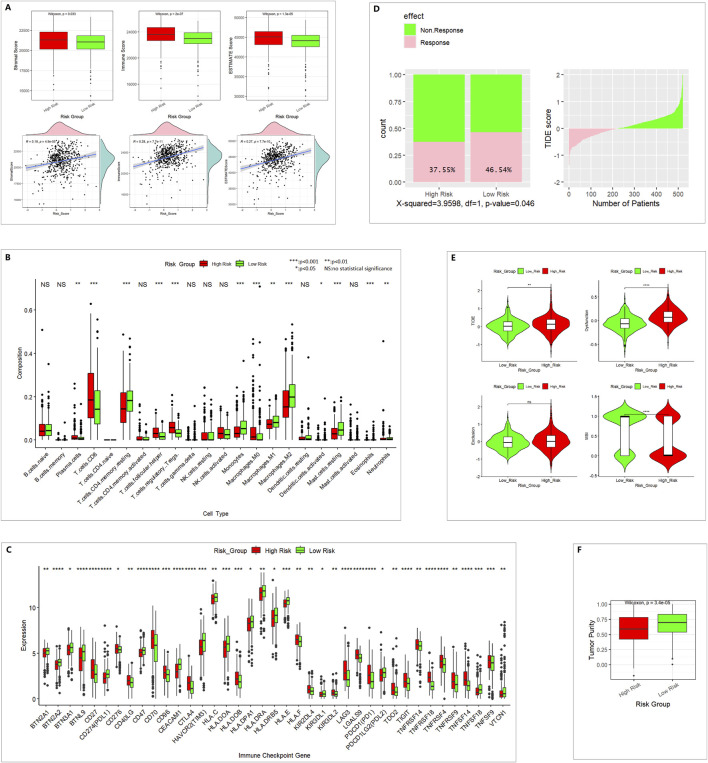
Different immune profiles between the low- and high-risk groups in the TCGA 521 ccRCC cohort. **(A)** ESTIMATE analysis for evaluating the infiltration of immune cells and stromal cells. **(B)** CIBERSORT analysis of 22 TIICs. **(C)** Of 79 ICGs, 39 showed a statistically significant difference. **(D)** TIDE predicted the proportion of patients with a response to immunotherapy. **(E)** TIDE analysis results. **(F)** Tumor purity.

In the high-risk group, we found higher stromal scores, immune scores, and ESTIMATE scores and lower tumor purity. The risk score and stromal score, immune score, and ESTIMATE score were positively correlated (Figures 5A,F). This means the infiltration of immune cells and stromal cells was higher in the high-risk group.

In 22 TIICs, the contents of regulatory T cells (Treg), M0 macrophages, and CD8^+^ T cells were remarkably higher in the high-risk group. The numbers of M1 macrophages, M2 macrophages, and monocytes were higher in the low-risk group (Figure 5B). M2 macrophages might be correlated with pro-tumor activity, whereas M1 macrophages might be correlated with antitumor activity. CD8^+^ T cells are the major antitumor cells. Treg cells can inhibit the anti-tumor immune effect (Dou and Fang, 2021; Peña-Romero and Orenes-Piñero, 2022). In summary, the difference between high- and low-risk groups in the tumor immune microenvironment was complex.

A total of 39 of 79 ICGs showed a statistically significant difference (Figure 5C). PDL1 and PDL2 were lower in the high-risk group, which indicates poor immunotherapy response.

In TIDE immunotherapy response prediction, the TIDE score and dysfunction score were higher in the high-risk group. The MSI (microsatellite instability) score was lower in the high-risk group (Figure 5E), and the response percentage was lower in the high-risk group (Figure 5D), which indicates poor immunotherapy response.

## 4 Discussion

MICALL2 is considered a typical cell mobility-promoting factor. Overexpression of MICALL2 could promote the proliferation, migration, and invasion of ccRCC cell lines (Zeng et al., 2023). FKBP10 knockdown could cause cell cycle arrest at the G0/G1 phase and reduce cell proliferation, invasion, and migration in 786-O and A-704 cell lines (Ge et al., 2017). FKBP10 might participate in the process of type I collagen synthesis in ccRCC via regulating crosslinking of pro-collagen chains (Zhang et al., 2021). FKBP10 might promote ccRCC progression and regulate sensitivity to HIF2α blockade by facilitating LDHA phosphorylation (Liu et al., 2024). ACADSB is involved in the metabolism of fatty acids and branched-chain amino acids and plays an important role in glioma, colorectal cancer, and hepatocellular carcinoma (Liu et al., 2022).

The mitochondrial-related gene signature based on MICALL2, FKBP10, and ACADSB could provide prognostic predictive value in ccRCC. The ccRCC patients in the high-risk group had an unfavorable prognosis. DEGs between the high- and low-risk groups were involved in pathways about extracellular-related function, immune-related function, or cytokine-mediated signaling pathways. Those pathways were associated with tumor invasion, tumor microenvironment, tumor immunotherapy response, or tumor metabolism. ESTIMATE analysis showed that the infiltration of immune cells and stromal cells was higher in the high-risk group. More Treg cells, fewer M1 macrophages, lower PDL1 expression levels, and lower PDL2 expression levels in the high-risk group might be associated with the poorer immunotherapy response. In addition, more elaborate analyses, such as single-cell data analyses, might be more informative. New methods of single-cell data analysis are showing potential (Yuan et al., 2024a; Yuan et al., 2024b; Yuan et al., 2025).

## 5 Conclusion

Our results suggested that our mitochondrial-related gene signature based on MICALL2, FKBP10, and ACADSB, as a risk model, could be a reliable ccRCC prognostic biomarker and could predict the response to immunotherapy. The risk score was correlated with the tumor microenvironment and immune cell infiltration.

## Data Availability

The datasets presented in this study can be found in online repositories. The names of the repository/repositories and accession number(s) can be found in the article/Supplementary Material.

## Supplementary material

SUPPLEMENTARY FIGURE S1 Flow chart of the work.

SUPPLEMENTARY FIGURE S2 Assessing the performance of the prognostic risk model by using more variable coefficient index values in the TCGA 521 ccRCC cohort. **(A)** K–M curve and ROC curve. **(B)** Distribution of risk score, survival status, and expression of three genes.

## Abbreviations

ccRCC, clear cell renal cell carcinoma; CI, confidence intervals; DEGs, differentially expressed genes; GO, Gene Ontology; ICGs, immune checkpoint genes; ICIs, immune checkpoint inhibitors; KEGG, Kyoto Encyclopedia of Genes and Genomes; MRGs, mitochondrial-related genes; MSI, microsatellite instability; OR, odds ratio; TIDE, tumor immune dysfunction and exclusion; TIICs, tumor-infiltrating immune cells; TKIs, tyrosine-kinase inhibitors.
