# Supplementary figures and images for: Constructing a novel mitochondrial-related gene signature for predicting survival and evaluating the tumor immune microenvironment in clear cell renal cell carcinoma
Source: Front Genet. 2025 Sep 22;16:1543593. doi: 10.3389/fgene.2025.1543593 (PMC12497594; doi:10.3389/fgene.2025.1543593)

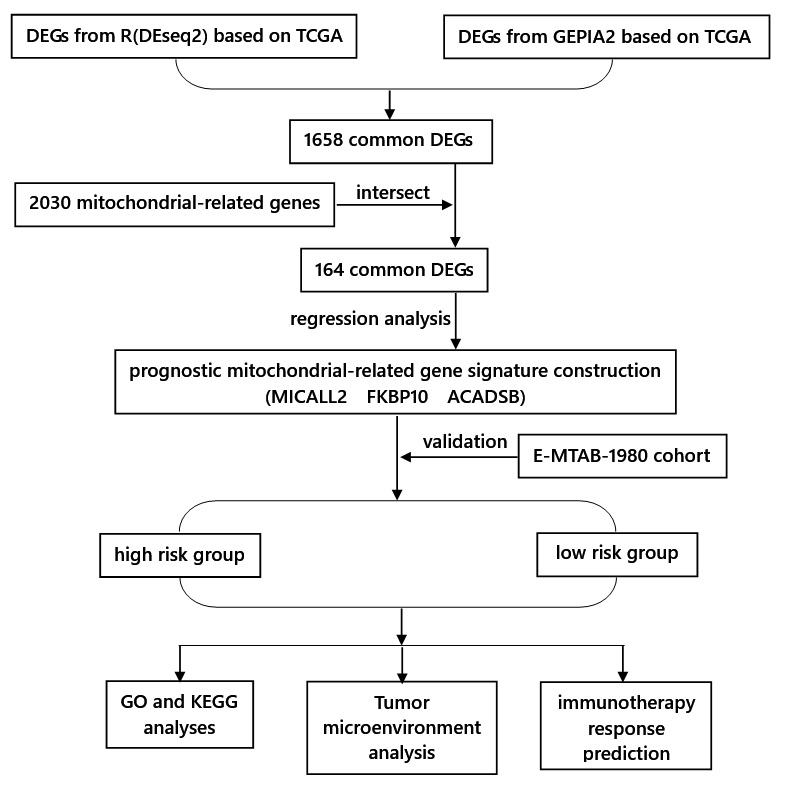

Supplement: Supplementary file 1 [file Image1.jpeg]

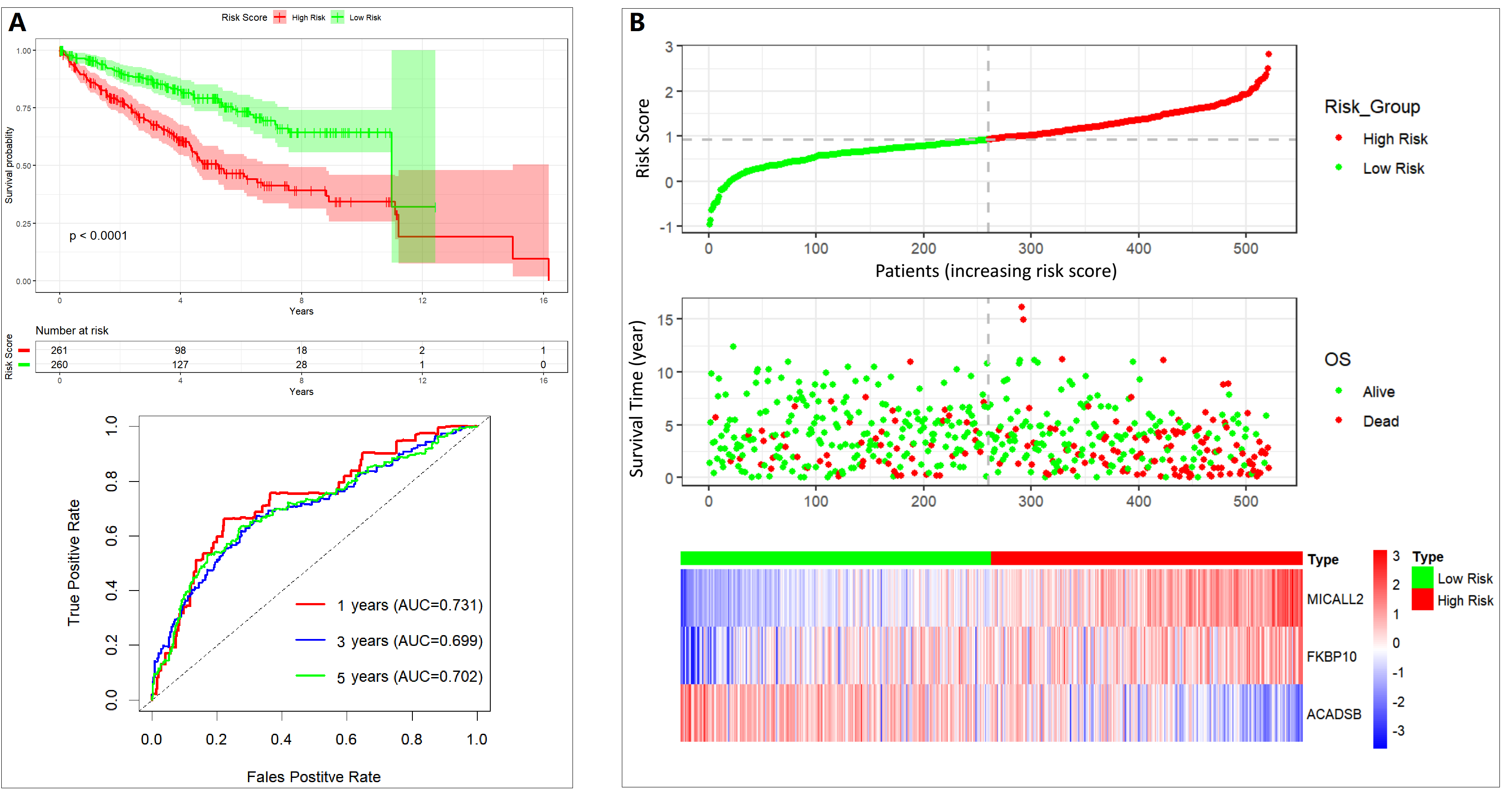

Supplement: Supplementary file 2 [file Image2.tif]
