# Supplementary material for: Constructing a novel mitochondrial-related gene signature for predicting survival and evaluating the tumor immune microenvironment in clear cell renal cell carcinoma
Source: Front Genet. 2025 Sep 22;16:1543593. doi: 10.3389/fgene.2025.1543593 (PMC12497594; doi:10.3389/fgene.2025.1543593)
Supplement: Supplementary file 3 [file DataSheet2.docx]

library(readr)

library(dplyr)

library(tidyverse)

library(tibble)

#install.packages("rjson")

library("rjson")

json <- jsonlite::fromJSON("metadata.cart.2024-01-18.json")

file_sample0 <- json[c('file_name','associated_entities')]

file_sample0$sample_id <- sapply(file_sample0$associated_entities,function(x){x[,1]})

file_sample <- subset(file_sample0, select = -associated_entities)

file_sample$file_name <- sapply(strsplit(file_sample$file_name,split='e_counts.tsv'),function(x){x[1]})

count_file <- list.files('gdc_download_20240215_183053.315287',pattern = '*rna_seq.augmented_star_gen',recursive = TRUE)

count_file_name <- strsplit(count_file,split='/')

count_file_name <- sapply(count_file_name,function(x){x[2]})

COUNT_Ensembl_matrix <- data.frame()

for (i in 1:length(count_file_name)){

path <- paste0('gdc_download_20240215_183053.315287//',count_file[i])

data0 <- read.table(path,fill = TRUE,header = TRUE)

data <-data0[-c(1:4),c(1,7)] #7提取TPM，4提取counts，8提取FPKM

colnames(data)[2] <- file_sample[which(file_sample$file_name == count_file_name[i]),'sample_id']

COUNT_Ensembl_matrix <- if (nrow(COUNT_Ensembl_matrix) == 0) data else merge(COUNT_Ensembl_matrix, data, by = "gene_id")

}

write.csv(COUNT_Ensembl_matrix,'TPM_Ensembl_matrix.csv',row.names = FALSE)

**将Ensembl ID矩阵转变为Gene Symbol矩阵（详细解说在“后续1”）：**

Ensembl_Symbol <- data0[c('gene_id','gene_name')]

COUNT_Symbol_matrix <- merge(Ensembl_Symbol,COUNT_Ensembl_matrix)

COUNT_Symbol_matrix <- COUNT_Symbol_matrix[-1]

COUNT_Symbol_matrix <- aggregate( . ~ gene_name,data=COUNT_Symbol_matrix, max)

rownames(COUNT_Symbol_matrix) <- COUNT_Symbol_matrix[,1]

COUNT_Symbol_matrix <- COUNT_Symbol_matrix[,-1]

write.csv(COUNT_Symbol_matrix,'TPM_Symbol_matrix.csv',row.names = TRUE)

#前面的data0矩阵就同时包含Gene Symbol列和Ensembl ID列。从data0矩阵中提取出这两列：

Ensembl_Symbol <- data0[c('gene_id','gene_name')]

COUNT_Symbol_matrix <- merge(Ensembl_Symbol,COUNT_Ensembl_matrix) #合并Ensembl_Symbol和COUNT_Ensembl_matrix两个矩阵

COUNT_Symbol_matrix <- COUNT_Symbol_matrix[-1] #得到Gene Symbol矩阵

#也可以用类似的方法从data0矩阵中提取出每个基因的基因类型（protein_coding/lncRNA/miRNA/rRNA等）

#Gene Symbol矩阵的gene_name列有重复，需要删除重复的基因，这里保留每个基因最大表达量结果

COUNT_Symbol_matrix <- aggregate( . ~ gene_name,data=COUNT_Symbol_matrix, max)

#将gene_name列设为行名

rownames(COUNT_Symbol_matrix) <- COUNT_Symbol_matrix[,1]

COUNT_Symbol_matrix <- COUNT_Symbol_matrix[,-1]

#write.csv(COUNT_Symbol_matrix,'COUNT_Symbol_matrix.csv',row.names = TRUE)

**后续2.分为normal和tumor矩阵**

--------------------------

sample <- colnames(COUNT_Symbol_matrix)

normal <- c()

tumor <- c()

for (i in 1:length(sample)){

if((substring(colnames(COUNT_Symbol_matrix)[i],14,15)>=10)){ #14、15位置大于等于10的为normal样本

normal <- append(normal,sample[i])

} else {

tumor <- append(tumor,sample[i])

}

}

tumor_matrix <- COUNT_Symbol_matrix[,tumor]

normal_matrix <- COUNT_Symbol_matrix[,normal]

#write.csv(tumor_matrix,'tumor_matrix.csv',row.names = TRUE)

#write.csv(normal_matrix,'normal_matrix.csv',row.names = TRUE)

**将TPM转变为log2（TPM+1）：**

library(dplyr)

tpm<-read.csv('TPM_Symbol_matrix.csv',row.names=1)

exp<-log2(tpm+1)

write.csv(exp,'log2TPMplus1_Symbol_matrix.csv',row.names = TRUE)

exp<-read.csv('log2TPMplus1_Symbol_matrix.csv')

geneFile41="41.txt"

geneRT=read.table(geneFile41,header = F,sep = "\t",check.names = F)

rownames(exp) <- exp[,1]

exp1=exp[as.vector(geneRT[,1]),]

write.csv(exp1,'41genelog2TPMplus1_Symbol_matrix.csv',row.names = TRUE)

TCGA.CJ.4639.01A.02R.1325.07 change to TCGA-CJ-4639-01A-02R-1325-07

exp1<-read.csv('41genelog2TPMplus1_Symbol_matrix.csv')

exp1=t(exp1)

write.csv(exp1,'41genelog2TPMplus1_Symbol_matrix.csv',row.names = FALSE)

exp1<-read.csv('41genelog2TPMplus1_Symbol_matrix.csv')

exp2<-read.csv('11selected_genes_wth_counts_with_clinical_606sample_NplusT.csv')

library(tidyverse)

expmix <- left_join(exp1, exp2, by="entity_submitter_id")

write.csv(expmix,'41_genes_wth_log2TPMplus1_with_clinical_NplusT.csv',row.names = FALSE)

**lasso分析+拉姆达图**

library(glmnet)
library(survival)
library(dplyr)

library(tidyverse)

lasso_data7<-read.csv('41_genes_wth_log2TPMplus1_with_clinical_Tumor521samples.csv')

# 把生存时间单位从day转换为year

#lasso_data$OS.time <- lasso_data$OS.time / 365

rownames(lasso_data7) <- lasso_data7[,1]

# 设置自变量和因变量
x <- as.matrix(lasso_data7[ , c(24:ncol(lasso_data7))])

#24 是从左往右数，第一个基因的列数，24：n就是从第24列到最后一列的意思
y <- as.matrix(Surv(lasso_data7$OS.time, lasso_data7$OS))

alpha1_fit <- glmnet(x, y, alpha = 1, family = "cox", nlambda = 100)

plot(alpha1_fit, xvar = "lambda", label = TRUE)

alpha1.fit.cv <- cv.glmnet(x, y, type.measure = "deviance", alpha = 1, family = "cox")

plot(alpha1.fit.cv)

print(alpha1.fit.cv)

coef(alpha1.fit.cv, s = alpha1.fit.cv$lambda.1se)

feature_all <- as.data.frame(as.matrix(coef(alpha1.fit.cv, s = alpha1.fit.cv$lambda.1se)))

colnames(feature_all) <- "coff"

feature_opt <- feature_all %>% filter(abs(coff) > 0)

rownames(feature_opt)

**多因素cox分析**

library(survival)
library(survminer)

mul_cox <- coxph(Surv(OS.time, OS) ~ age_at_index + ethnicity + gender + race+ajcc_pathologic_stage+ALDH6A1+FKBP10+MICALL2+ACADSB, data = lasso_data7)

summary(mul_cox)

#提取p和HR

x <- summary(mul_cox)

pvalue <- signif(as.matrix(x$coefficients)[ , 5], 2)

HR <- signif(as.matrix(x$coefficients)[ , 2], 2)

low <- signif(x$conf.int[ , 3], 2)

high <- signif(x$conf.int[ , 4], 2)

res_mul_cox <- data.frame(p.value = pvalue, HR = paste0(HR, " (", low, "-", high, ")", sep = ""), stringsAsFactors = F)

res_mul_cox

# Significance Codes as ***:p<0.001; **:p<0.01; *:p<0.05; .:p<0.1

#画森林图

ggforest(mul_cox, data = lasso_data7)

**利用mutiCOX的coef 或ggrisk的coef，构建预后模型**

**--风险图：**

#install.packages("ggrisk")

library(ggrisk)

library(survival)

library(rms)

fit <- cph(Surv(OS.time,OS)~FKBP10+MICALL2+ACADSB,lasso_data9)

cph(Surv(OS.time,OS)~FKBP10+MICALL2+ACADSB,lasso_data9)

**（factorY）**Risk score = 0.1995*FKBP10 + 0.3089*MICALL2 - 0.4429*ACADSB.

lasso_data9<-read.csv('41_genes_wth_log2TPMplus1_with_clinical_Tumor521samples.csv')

rownames(lasso_data9) <- lasso_data9[,1]

lasso_data9$FKBP10p<-lasso_data9$FKBP10*0.1995

lasso_data9$MICALL2p<-lasso_data9$MICALL2*0.3089

lasso_data9$ACADSBp<-lasso_data9$ACADSB*0.4429

#计算所得结果新列一列

lasso_data9$factorY<-lasso_data9$FKBP10p+lasso_data9$MICALL2p-lasso_data9$ACADSBp

write.csv(lasso_data9,'41_genes_wth_log2TPMplus1_with_clinical_Tumor_with_factorX_factorY_calculated.csv',row.names = FALSE)

ggrisk(fit,

cutoff.value='median',

cutoff.x = 145,

cutoff.y = -0.8

)

lasso_data10<-read.csv('41_genes_wth_log2TPMplus1_with_clinical_Tumor_with_factorX_calculatedfuben.csv',row.names=1)

fit <- cph(Surv(OS.time,OS)~FKBP10+MICALL2+ACADSB,lasso_data10)

cph(Surv(OS.time,OS)~FKBP10+MICALL2+ACADSB,lasso_data10)

ggrisk(fit,

cutoff.value='median',

cutoff.x = 145,

cutoff.y = -0.8,

title.B.ylab='Survival Time(year)'

)

**risk三联图**

#install.packages("pheatmap")

library(ggplot2)

library(pheatmap)

library(ggplotify)

library(cowplot)

lasso_data9<-read.csv('41_genes_wth_log2TPMplus1_with_clinical_Tumor_with_factorX_factorY_calculated.csv')

rownames(lasso_data9) <- lasso_data9[,1]

lasso_data9$Risk_Group <- ifelse(lasso_data9$factorY<median(lasso_data9$factorY),'Low Risk','High Risk')

#将风险评分按中位数拆分为高/低风险两组

lasso_data9 <- lasso_data9[order(lasso_data9$factorY,decreasing = F),]

#按风险评分从低到高排序

lasso_data9$id <- c(1:length(lasso_data9$factorY))

#根据调整后顺序建立编号id

p1 <- ggplot(lasso_data9,aes(x = id,y = factorY)) +

geom_point(aes(col = Risk_Group)) +

scale_colour_manual(values = c("red","green")) +

geom_hline(yintercept = median(lasso_data9$factorY), colour="grey", linetype="dashed", linewidth=0.8) +

geom_vline(xintercept = sum(lasso_data9$Risk_Group == "Low Risk"), colour="grey", linetype = "dashed", linewidth = 0.8) +

theme_bw()

p1

p2 <- ggplot(lasso_data9,aes(x = id,y = OS.time)) +

geom_point(aes(col = OS)) +

scale_colour_manual(values = c("green","red")) +

geom_vline(xintercept = sum(lasso_data9$Risk_Group == "Low Risk"), colour = "grey", linetype = "dashed", linewidth = 0.8) +

theme_bw()

p2

lasso_data9<-read.csv('41_genes_wth_log2TPMplus1_factorY_calculatedOSchange.csv',row.names=1)

将p1和p2对齐合并，热图后续再word里微调对齐

plot_grid(p1,p2, nrow = 2,align = "v", axis = "tlbr")

**p3热图绘制：**

lasso_data11<-read.csv('41_genes_wth_log2TPMplus1_with_clinical_Tumor_with_factorX_factorY_calculated.csv')

rownames(lasso_data11) <- lasso_data11[,1]

mycol <- colorRampPalette(c("blue","white","red"))(100)

#自定义颜色

exp11 <- lasso_data11[,c(61,38,62)]

#将三个基因按高低风险单列

exp12 <- t(scale(exp11))

#行列对调

#添加分组信息：

annotation <- data.frame(Type = as.vector(lasso_data11[,73]))

rownames(annotation) <- colnames(exp12)

annotation$Type <- factor(annotation$Type,levels = c('Low Risk','High Risk'))

head(annotation)

ann_colors <- list(Type = c('Low Risk' = "green",

'High Risk' = "red"))

pheatmap(exp12,

col= mycol,

cluster_rows = F,

cluster_cols = F,

show_colnames = F,

annotation_col = annotation,

annotation_colors = ann_colors,

annotation_legend = T

)

**（factorX）**Risk score = 0.127374*FKBP10 + 0.379045*MICALL2 - 0.249544*ACADSB.

lasso_data9<-read.csv('41_genes_wth_log2TPMplus1_with_clinical_Tumor521samples.csv')

rownames(lasso_data9) <- lasso_data9[,1]

lasso_data9$FKBP10plus<-lasso_data9$FKBP10*0.127374

lasso_data9$MICALL2plus<-lasso_data9$MICALL2*0.379045

lasso_data9$ACADSBplus<-lasso_data9$ACADSB*0.249544

#计算所得结果新列一列

lasso_data9$factorX<-lasso_data9$FKBP10plus+lasso_data9$MICALL2plus-lasso_data9$ACADSBplus

write.csv(lasso_data9,'41_genes_wth_log2TPMplus1_with_clinical_Tumor_with_factorX_calculated.csv',row.names = FALSE)

**---做KM曲线**

library(survminer)

library(survival)

lasso_data14<-read.csv('41_genes_wth_log2TPMplus1_factorY_calculatedOSchange.csv',row.names=1)

fit <- survfit(Surv(OS.time, OS) ~ Risk_Group, data = lasso_data14)

fit

ggsurvplot(

fit,

data = lasso_data14,

censor = T,

censor.shape = "|", censor.size = 4,

conf.int = TRUE,

conf.int.style = "ribbon",

conf.int.alpha = 0.3,

pval = TRUE,

pval.size = 5,

legend = "top",

legend.title = 'Risk Score',

legend.labs = c("High Risk","Low Risk"),

xlab = "Years",

ylab = "Survival probablity",

palette = c('red','green'),

ggtheme = theme_bw(),

risk.table = TRUE,

risk.table.col = "black",

risk.table.title = 'Number at risk',

fontsize = 4,

risk.table.y.text = FALSE,

risk.table.height = 0.2,

)

**---做ROC-AUC**

#install.packages("mice")

library(survival)

library(survminer)

library(survivalROC)

library(mice)

library(pROC)

lasso_data15<-read.csv('41_genes_wth_log2TPMplus1_factorXOSchange.csv',row.names=1)

cutoff=1

cutoff1=3

cutoff2=5

ROC <- survivalROC(Stime = lasso_data15$OS.time,

status = lasso_data15$OS,

marker = lasso_data15$factorX,

predict.time = cutoff,

method = 'KM')

plot(ROC$FP,ROC$TP,type = 'l',col='red',xlim = c(0,1),ylim = c(0,1),lwd=3,

xlab = 'Fales Postitve Rate',ylab = 'True Positive Rate')

abline(0,1,col='black',lty=2)

aucText1=paste0("1 years"," (AUC=",sprintf("%.3f",ROC$AUC),")")

ROC1 <- survivalROC(Stime = lasso_data15$OS.time,

status = lasso_data15$OS,

marker = lasso_data15$factorX,

predict.time = cutoff1,

method = 'KM')

lines(ROC1$FP, ROC1$TP, type="l", xlim=c(0,1), ylim=c(0,1),col="blue",lwd = 2)

aucText2=paste0("3 years"," (AUC=",sprintf("%.3f",ROC1$AUC),")")

ROC2 <- survivalROC(Stime = lasso_data15$OS.time,

status = lasso_data15$OS,

marker = lasso_data15$factorX,

predict.time = cutoff2,

method = 'KM')

lines(ROC2$FP, ROC2$TP, type="l", xlim=c(0,1), ylim=c(0,1),col="green",lwd = 2)

aucText3=paste0("5 years"," (AUC=",sprintf("%.3f",ROC2$AUC),")")

legend("bottomright", c(aucText1,aucText2,aucText3),

lwd=2,bty="n",col=c("red","blue","green"),cex=1)

**四格表**

**---第一个表**

#install.packages("gtsummary")

library(gtsummary)

lasso_data17<-read.csv('041_genes_wth_log2TPMplus1_with_clinical_Tumor_with_factorYplus_final_final_final.csv',row.names=1)

lasso_data18 <- lasso_data17 %>% dplyr::select(Age,Gender,Race,Ethnicity,T_Stage,N_Stage,M_Stage,Tumor_Stage,Survival_Status,Risk_Group)

**改成NA加missing：**

lasso_data18 %>%

tbl_summary(

by = Risk_Group,

type = all_continuous() ~ "continuous2",

statistic = all_continuous() ~ c("{mean} ({sd})",

"{median} ({p25}, {p75})",

"{min}, {max}"),

missing="always",

missing_text='missing')%>%

add_p(pvalue_fun = ~style_pvalue(.x, digits = 2))

**---第二个表**

library(gtsummary)

library(survival)

library(gtsummary)

lasso_data17<-read.csv('041_genes_wth_log2TPMplus1_with_clinical_Tumor_with_factorYplus_final_final_final.csv',row.names=1)

lasso_data18 <- lasso_data17 %>% dplyr::select(Age,Gender,Race,Ethnicity,T_Stage,N_Stage,M_Stage,Tumor_Stage,Survival_Status,OS.time,Risk_Group)

t2 <-coxph(Surv(OS.time, Survival_Status) ~ Age+Gender+Race+Ethnicity+T_Stage+N_Stage+M_Stage+Tumor_Stage+Risk_Group,lasso_data18)%>%

tbl_regression(exponentiate = TRUE)

**带误差线的点阵图**

#install.packages("ggbeeswarm")

library(tidyverse)

library(ggbeeswarm)

lasso_data8<-read.csv('41_genes_wth_log2TPMplus1_with_clinical_NplusT592samples.csv')#肿瘤和正常都有

rownames(lasso_data8) <- lasso_data8[,1]

ggplot(data=lasso_data8,mapping=aes(x=condition,y=ACADSB))+

geom_dotplot(data=lasso_data8,mapping=aes(x=condition,y=ACADSB,fill=condition),

binaxis="y",stackdir="center",binwidth=1/8)+

scale_fill_manual(values = c("blue", "red"))+

stat_summary(fun.data="mean_sdl",fun.args=list(mult=1),geom="errorbar",

color="black",width=0.2)+

stat_summary(fun="mean",fun.args=list(mult=1),geom="crossbar", linewidth=0.2,width=0.8,

color="black",size=4)+

scale_x_discrete(name=" ",labels=c("Normal","Tumor"))+

guides(fill="none")+

theme_light()+

theme(axis.text=element_text(color="black"))

**计算P值加进图里**

lasso_data8<-read.csv('41_genes_wth_log2TPMplus1_with_clinical_NplusT592samples.csv')#肿瘤和正常都有

rownames(lasso_data8) <- lasso_data8[,1]

t.test(MICALL2 ~ condition, data = lasso_data8, paired=F)

**免疫浸润**

install.packages("e1071")

install.packages("parallel")

install.packages('BiocManager')

library(BiocManager)

BiocManager::install('preprocessCore')

install.packages("devtools")

library(devtools)

devtools::install_github('shenorrlab/bseqsc')

library(bseqsc)

library(e1071)

library(parallel)

library(preprocessCore)

source('04Cibersort.R')

04LM22.file <- "04LM22.txt "

04TPM_Symbol_matrix.file <- "04TPM_Symbol_matrix.txt "

immune <- CIBERSORT('04LM22.file','04TPM_Symbol_matrix.file', perm = 1000, QN = T)

write.table(immune, "immune.txt",

sep = "\t", row.names = T, col.names = T, quote = F)

immuneplus<-read.csv('04factorYplus_final_final_final_for_immune.csv')

rownames(immuneplus) <- immuneplus [,1]

write.csv(immune,'immune.csv',row.names = T)

immune1<-read.csv('immune.csv')

rownames(immune1) <- immune1 [,1]

library(tidyverse)

immune2 <- left_join(immune1, immuneplus, by="entity_submitter_id")

write.csv(immune2,'immune2.csv',row.names = F)

library(tidyverse)

bbb<-read.csv('04factorYplus_final_final_final_for_immuneless.csv')

immune3<-read.csv('immune2less.csv')

class(bbb$Risk_Group)

immune3$Risk_Group <- bbb$Risk_Group

library(ggsci)

library(tidyr)

library(ggpubr)

bbb <- gather(immune3,key=Cell_Type,value = Composition,-c(Risk_Group,entity_submitter_id))

**ggboxplot(bbb, x = "Cell_Type", y = "Composition",**

**fill = "Risk_Group", color = "black", palette = c("red", "green"))+**

**stat_compare_means(aes(group = Risk_Group),**

**method = "****wilcox.test",**

**label = "p.signif",**

**symnum.args=list(cutpoints = c(0, 0.001, 0.01, 0.05, 1),**

**symbols = c("***","**","*","NS")))+**

**theme(text = element_text(size=10),**

**axis.text.x = element_text(angle=45, hjust=1))**

**TIDE预测免疫疗效，**

library(stringr)

library(ggplot2)

library(ggpubr)

library(patchwork)

TIDE<-read.csv('04TPM_Symbol_matrix.csv',row.names=1)

TIDE=t(TIDE)

library(tidyverse)

immune4<-read.csv('04factorYplus_final_final_final_for_immuneless1.csv')

write.csv(TIDE,'TIDE.csv',row.names = F)

TIDE<-read.csv('TIDE.csv')

TIDE1 <- left_join(immune4, TIDE, by="entity_submitter_id")

write.csv(TIDE1,'TIDE1.csv',row.names = F)

rownames(TIDE1) <- TIDE1 [,2]

TIDE1 <- TIDE1[, -2]#删除第二列

TIDE1 <- TIDE1[, -1]

TIDE1=t(TIDE1)

TIDE2 <- t(apply(TIDE1, 1, function(x){x-(mean(x))})) #均值标准化处理

write.table(TIDE2, "TIDE2.txt", sep = "\t", row.names = T)

#保存成row.name为第一列的制表符（\）分隔的txt文件

**小提琴图4个：**

TIDE3 <- data.table::fread('TIDE_net_calculate_result.csv')

TIDE3$Risk_Group <- ifelse(

str_sub(TIDE3$Patient,1,1)=='L','Low_Risk','High_Risk'

)

TIDE3$Risk_Group <- factor(TIDE3$Risk_Group,levels = c('Low_Risk','High_Risk'))

my_comparisons <- list( c("Low_Risk", "High_Risk"))

p1 <- ggviolin(TIDE3, x = 'Risk_Group', y = 'TIDE', fill = 'Risk_Group',

palette = c("green","red"),

add = 'boxplot', add.params = list(fill = "white")) +

stat_compare_means(comparisons = my_comparisons, label = "p.signif", bracket.size=0.5, tip.length = 0.02, method = 't.test')

p1 #保存尺寸484-450 #method可选：t.test、wilcox.test、anova、kruskal.test

Dysfunction、Exclusion就是把TIDE换一下。

MSI要搞一下：

colnames(TIDE3)[6] <- c('MSI')

其他都一样，就MSI把TIDE换一下。

**图片多张拼一张：**

library(patchwork)

> p0 <- (p1+p2+p3+p4)+plot_annotation(tag_levels = 'A')

> p0

**免疫疗效预测 条形图1个：**

dat_plot3 <- data.frame(id3 = TIDE3$Patient,

t3 = TIDE3$TIDE)

**dat_plot3$threshold3 = factor(ifelse(dat_plot3$t3 < 0,'Response','Non.Response'),levels=c('Response','Non.Response'))**

dat_plot3 <- dat_plot3 %>% arrange(t3)

dat_plot3$id3 <- factor(dat_plot3$id3,levels = dat_plot3$id3)

library(ggplot2)

library(ggthemes)

library(ggprism)

p5 <- ggplot(data = dat_plot3,aes(x = id3,y = t3,fill = threshold3)) +

geom_col()+

scale_x_continuous(limits = c(0,522))+

scale_fill_manual(values = c('**Response**'= 'pink','**Non.Response**'='green')) +

geom_hline(yintercept = c(-2,2),color = 'white',size = 0.5,lty='dashed') +

xlab('Number of Patients') +

ylab('TIDE score') +

guides(fill=guide_legend(key.linewidth = 3, key.height = 5, nrow = 2, ncol = 1, byrow = TRUE))

write.csv(dat_plot3,'dat_plot3.csv',row.names = F)

手动修修数据

colnames(dat_plot3)[3] <- c('effect')

dat_plot4<-read.csv('04factorYplus_final_final_final_for_immuneless1.csv')

dat_plot5<-read.csv('04factorYplus_final_final_final_for_immuneless.csv')

dat_plot4 <- left_join(dat_plot4, dat_plot3, by="patient")

dat_plot5 <- left_join(dat_plot5, dat_plot4, by="entity_submitter_id")

table(dat_plot5$Risk_Group[dat_plot5$effect=="Response"])

High Risk Low Risk

98 121

table(dat_plot5$Risk_Group[dat_plot5$effect=="Non.Response"])

High Risk Low Risk

163 139

Risk_Group <- c("Low Risk","Low Risk","High Risk","High Risk")

effect <- c("Non.Response","Response","Non.Response","Response")

num <- c(139,121,163,98)

Percent <- c(0.5346,0.4654,0.6245,0.3755)

data66 <- data.frame(Risk_Group,effect,Percent,num)

Non.Response <- c(139,163)

Response <- c(121,98)

dat66 <- data.frame(Non.Response,Response)

rownames(dat66) <- c("Low Risk","High Risk")

chisq.test(dat66)

结果：

Pearson's Chi-squared test with Yates' continuity correction

data: dat66

X-squared = 3.9598, df = 1, p-value = 0.0466

p6 = ggplot( data66, aes( x = Risk_Group, weight = Percent, fill = effect))+

scale_fill_manual(values = c('Response'= 'pink','Non.Response'='green')) +

geom_bar( position = "stack")+xlab("X-squared=3.9598, df=1, p-value=0.0466")+guides(fill = "none")#去掉图例

p7 <- (p6+p5)+plot_annotation(tag_levels = 'A')

**肿瘤基质score、纯度等4个图和4个相关性图：**

**（参考**[**https://www.jianshu.com/p/0402df10f6f5**](https://www.jianshu.com/p/0402df10f6f5)**等）**

estimate1<-read.csv('04log2TPMplus1_Symbol_matrix_tumor521.csv',row.names=1)

estimate1=t(estimate1)

estimate2<-read.csv('04factorYplus_final_final_final_for_immuneless.csv')

library(tidyverse)

write.csv(estimate1,'estimate1.csv',row.names = F)

estimate1<-read.csv('estimate1.csv')

estimate1 <- left_join(estimate2, estimate1, by="entity_submitter_id")

rownames(estimate1) <- estimate1 [,1]

estimate1 <- estimate1 [, -2]#删除第二列

estimate1 <- estimate1 [, -1]

estimate1=t(estimate1)

library(utils)

rforge <-"http://r-forge.r-project.org"

install.packages("estimate",repos=rforge, dependencies=TRUE)

library(estimate)

write.table(estimate1, file = "estimate1.txt", sep = '\t', quote = F)

estimate3 = "estimate1.txt"

in.gct.file = "ESTIMATE_input.gct"

outputGCT(estimate3, in.gct.file)

out.score.file = "ESTIMATE_score.gct"

estimateScore(in.gct.file,

out.score.file,

platform="illumina") # TCGA数据,选择illumina

ESTIMATE_score = read.table(out.score.file,

skip = 2,

header = T,

row.names = 1)

write.csv(ESTIMATE_score,'ESTIMATE_score.csv',row.names = F)

ESTIMATE_score<-read.csv('ESTIMATE_score.csv',row.names=1)

ESTIMATE_score=t(ESTIMATE_score)

write.csv(ESTIMATE_score,'ESTIMATE_score.csv',row.names = F)

ESTIMATE_score<-read.csv('ESTIMATE_score.csv')

ESTIMATE_score <- left_join(estimate2, ESTIMATE_score, by="entity_submitter_id")

用13年NC文章中的公式计算肿瘤纯度。

参考（https://www.jianshu.com/p/0b1de1427458）

公式是：Tumour purity=cos (0.6049872018+0.0001467884 × ESTIMATE score)

ESTIMATE_score$TumorPurity<-cos(0.6049872018+ESTIMATE_score$ESTIMATEScore*0.0001467884)

#计算所得结果新列一列

write.csv(ESTIMATE_score,'ESTIMATE_score_final.csv',row.names = F)

library(ggpubr)

library(ggsci)

library(patchwork)

p1 <- ggplot(ESTIMATE_score, aes(x = Risk_Group, y = StromalScore, fill = Risk_Group)) +

geom_boxplot(position = position_dodge(0.8)) +

scale_fill_manual(values = c('Low Risk'= 'green','High Risk'='red')) +

labs(x = "Risk Group", y = 'Stromal Score') +

stat_compare_means() +

theme_bw(base_size = 16) +

theme(axis.text.x = element_text(angle = 30,vjust = 0.85,hjust = 0.75),

legend.position = 'none')

画关联图

（参考<https://mp.weixin.qq.com/s/6NUUd_TKYd9QwLzl6AnMuQ>）

library(ggplot2)

library(ggpubr)

library(ggExtra)

estimate4<-read.csv('04factorYplus_final_final_final_for_immuneless2.csv')

ESTIMATE_score3 <- left_join(estimate4, ESTIMATE_score, by="entity_submitter_id")

rownames(ESTIMATE_score3) <- ESTIMATE_score3 [,1]

ESTIMATE_score3=t(ESTIMATE_score3)

write.table(ESTIMATE_score3, "ESTIMATE_score3.txt",

sep = "\t", row.names = T, col.names = T, quote = F)

inputFile="ESTIMATE_score3.txt"

Risk_Score="Risk_Score"

StromalScore="StromalScore"

rt=read.table(inputFile,sep="\t",header=T,check.names=F,row.names=1)

x=as.numeric(rt[Risk_Score,])

y=as.numeric(rt[StromalScore,])

df1=as.data.frame(cbind(x,y))

corT=cor.test(x,y,method="spearman")

cor=corT$estimate

pValue=corT$p.value

p11=ggplot(df1, aes(x, y)) +

xlab(Risk_Score)+ylab(StromalScore)+

geom_point()+ geom_smooth(method="lm",formula = y ~ x) + theme_bw()+

stat_cor(method = 'spearman', aes(x =x, y =y))

p11

p111=ggMarginal(p11, type = "density", xparams = list(fill = "pink"),yparams = list(fill = "#9fd7d3"))

p111

ImmuneScore="ImmuneScore"

x=as.numeric(rt[Risk_Score,])

y=as.numeric(rt[ImmuneScore,])

df1=as.data.frame(cbind(x,y))

corT=cor.test(x,y,method="spearman")

cor=corT$estimate

pValue=corT$p.value

p22=ggplot(df1, aes(x, y)) +

xlab(Risk_Score)+ylab(ImmuneScore)+

geom_point()+ geom_smooth(method="lm",formula = y ~ x) + theme_bw()+

stat_cor(method = 'spearman', aes(x =x, y =y))

p222=ggMarginal(p22, type = "density", xparams = list(fill = "pink"),yparams = list(fill = "#9fd7d3"))

ESTIMATEScore="ESTIMATEScore"

x=as.numeric(rt[Risk_Score,])

y=as.numeric(rt[ESTIMATEScore,])

df1=as.data.frame(cbind(x,y))

corT=cor.test(x,y,method="spearman")

cor=corT$estimate

pValue=corT$p.value

p33=ggplot(df1, aes(x, y)) +

xlab(Risk_Score)+ylab(ESTIMATEScore)+

geom_point()+ geom_smooth(method="lm",formula = y ~ x) + theme_bw()+

stat_cor(method = 'spearman', aes(x =x, y =y))

p333=ggMarginal(p33, type = "density", xparams = list(fill = "pink"),yparams = list(fill = "#9fd7d3"))

TumorPurity=" TumorPurity"

x=as.numeric(rt[Risk_Score,])

y=as.numeric(rt[TumorPurity,])

df1=as.data.frame(cbind(x,y))

corT=cor.test(x,y,method="spearman")

cor=corT$estimate

pValue=corT$p.value

p44=ggplot(df1, aes(x, y)) +

xlab(Risk_Score)+ylab(TumorPurity)+

geom_point()+ geom_smooth(method="lm",formula = y ~ x) + theme_bw()+

stat_cor(method = 'spearman', aes(x =x, y =y))

p444=ggMarginal(p44, type = "density", xparams = list(fill = "pink"),yparams = list(fill = "#9fd7d3"))

结果：Error in cor.test.default(x, y, method = "spearman") : 有限值的观察量不够，即结果为null

**免疫检查点基因：**

79个基因

ICE1<-read.csv('04log2TPMplus1_Symbol_matrix_tumor521.csv')

gene79="79.txt"

geneRT79=read.table(gene79,header = T,sep = "\t",check.names = F)

rownames(ICE1) <- ICE1 [,1]

rownames(geneRT79) <- geneRT79 [,1]

geneRT79plus=ICE1[as.vector(geneRT79[,1]),]

**11个缺失没测到，剩68个**

ICE2<-read.csv('geneRT79plus.csv',row.names = 1)

ICE2=t(ICE2)

write.csv(ICE2,'ICE2.csv',row.names = F)

ICE3<-read.csv('ICE2.csv')

rownames(ICE3) <- ICE3 [,1]

ICE4<-read.csv('04factorYplus_final_final_final_for_immuneless.csv')

rownames(ICE4) <- ICE4 [,1]

library(tidyverse)

ICE5 <- left_join(ICE4, ICE3, by="entity_submitter_id")

write.csv(ICE5,'ICE_finalfinalfinal.csv',row.names = F)

ICE6<-read.csv('ICE_finalfinalfinal.csv')

library(ggpubr)

library(ggplot2)

library(tidyverse)

library(reshape2)

library(ggsci)

rownames(ICE6) <- ICE6 [,1]

ICE7 <- gather(ICE6,key=Immune_Checkpoint_Gene,value = Expression,-c(Risk_Group,entity_submitter_id))

write.csv(ICE7,'ICE7.csv',row.names = F)

ICE8<-read.csv('ICE7.csv')

**p1<- ggplot(ICE8, aes(x = Immune_Checkpoint_Gene, y =Expression ))+**

**labs(y="Expression",x= "Immune Checkpoint Gene")+**

**geom_boxplot(aes(fill = Risk_Group),position=position_dodge(0.5),width=0.5)+**

**scale_fill_manual(values = c('Low Risk'= 'green','High Risk'='red')) +**

**theme_classic() +**

**theme(axis.title = element_text(size = 10,color ="black"),**

**axis.text = element_text(size= 10,color = "black"),**

**panel.grid.minor.y = element_blank(),**

**panel.grid.minor.x = element_blank(),**

**axis.text.x = element_text(angle = 45, hjust = 1 ),**

**panel.grid=element_blank(),**

**legend.position = "top",**

**legend.text = element_text(size= 10),**

**legend.title= element_text(size= 10)**

**) +**

**stat_compare_means(aes(group = Risk_Group),**

**label = "p.signif",#添加星号标签**

**method = "t.test",#kruskal.test多组检验,注意根据数据类型选择对的检验法**

**hide.ns = F)**#显示不显著的

**go、kegg、gsea三联圈图**

circle1<-read.csv('COUNT_Symbol_matrix_less.csv',row.names = 1)

circle1=t(circle1)

write.csv(circle1,'circle1.csv',row.names = F)

circle2<-read.csv('circle1.csv')

circle0<-read.csv('04factorYplus_final_final_final_for_immuneless.csv')

library(tidyverse)

circle_all <- left_join(circle0, circle2, by="entity_submitter_id")

write.csv(circle_all,'circle_all.csv',row.names = F)

拆分成high组和low组

circle_all_high_risk <- circle_all[circle_all$Risk_Group == "High Risk", ]

circle_all_low_risk <- circle_all[circle_all$Risk_Group == "Low Risk", ]

rownames(circle_all_low_risk) <- circle_all_low_risk [,1]

circle_all_low_risk <- circle_all_low_risk [, -2]#删除第二列

circle_all_low_risk <- circle_all_low_risk [, -1]

rownames(circle_all_high_risk) <- circle_all_high_risk [,1]

circle_all_high_risk <- circle_all_high_risk [, -2]#删除第二列

circle_all_high_risk <- circle_all_high_risk [, -1]

circle_all_high_risk=t(circle_all_high_risk)

circle_all_low_risk=t(circle_all_low_risk)

**data_circle <- merge(circle_all_high_risk,circle_all_low_risk, by = "row.names", all = TRUE)**

View(data_circle)

rownames(data_circle) <- data_circle$Row.names

data_circle <- data_circle[-1]

**【#这第一句和下面一句一个效果**

【#rownames(circle_all_high_risk) <- circle_all_high_risk [,1]

zero_counts_circle <- apply(data_circle == 0, 1, sum)

data_circle1 <- data_circle[-which(zero_counts_circle>0),]

#仅保留那些在所有样本中都表达的基因

group_list_circle <- c(rep('circle_all_high_risk',ncol(circle_all_high_risk)),rep('circle_all_low_risk',ncol(circle_all_low_risk)))

condition_circle <- factor(group_list_circle)

coldata_circle <- data.frame(row.names = colnames(data_circle1), condition_circle)

library(DESeq2)

dds_circle <- DESeqDataSetFromMatrix(countData = data_circle1, colData = coldata_circle, design = ~ condition_circle)

dds_circle_final <- DESeq(dds_circle)

result_circle <- as.data.frame(results(dds_circle_final))

DGE_circle <-subset(result_circle,padj < 0.05 & (log2FoldChange > 1 | log2FoldChange < -1))

DGE_circle <- DGE_circle[order(DGE_circle$log2FoldChange),]

write.csv(DGE_circle,'DGE_circle.csv',row.names = TRUE)

circle3<-read.csv('11go_and_kegg.csv')

library(ggplot2)

p9 <- ggplot(circle3,aes(Fold.Enrichment, fct_reorder(factor(Term), Fold.Enrichment))) +

geom_point(aes(size=Count,color=FDR,shape=Category)) +

scale_color_gradient(low="red",high = "green") +

labs(color="P.adj value",size="Count", shape=" ",

x="Fold Enrichment",y="Term",title="GO and KEGG enrichment") +

theme_bw()

p9

p9 + facet_wrap( ~ Category,ncol= 1,scale='free')

整circle图：

circle4<-read.csv('03DGE_circle_less.csv')

genelist9 <- data.frame(ID9 = circle4$gene, logFC9 = circle4$logFC)

GOterms9<-read.csv('03go_and_kegg_less.csv')

install.packages("GOplot")

library(GOplot)

GOChord(chord, limit = c(3, 0), gene.order = 'logFC') #只画参与3个以上进程的基因

library(stringr)

library(dplyr)

library(tidyr)

library(reshape2)

dt9 <- as_tibble(GOterms9[c(2,10)])

dt9$Term <- as.factor(dt9$Term)

df9 <- str_split(dt9$Genes,", ",n=16,simplify=TRUE)

dt9plus <- data.frame(dt9[,1],df9)

for (i in 2:17) {

dt9plus[,i] <- str_replace_all(dt9plus[,i], pattern="^$", NA_character_)

}

dt9final <- pivot_longer(dt9plus,!Term, names_to = "index",

values_to = "gene",

values_drop_na = TRUE)

n <- nrow(dt9final)

dt9final$value <- rep(1,n)

dt9finalplus <- dt9final[-2]

mat9 <- acast(dt9finalplus,gene~Term)

mat9[is.na(mat9)] <- 0

ind <- row.names(mat)

rownames(circle4) <- circle4 [,1]

logFC <- circle4[ind9,]$logFC

mat9final <- data.frame(mat9,logFC)

#这俩黑色的命令提不出来log FC值，就换另一种语句。

write.csv(mat9,'mat9.csv',row.names = TRUE)

mat9<-read.csv('mat9.csv')

rownames(mat9) <- mat9 [,1]

library(tidyverse)

mat9final <- left_join(mat9, circle4, by="gene")

#还是不行，后面发现是gene名字里有空格。。。

mat9final<-read.csv('mat9final.csv',row.names = 1)

library(GOplot)

GOChord(mat9final,space=0.02,

gene.size=2.5,

process.label=9,

border.size=0.1,

#limit = c(2, 0), #只画参与2个以上进程的基因，建议gene少的时候可以不限制这个

gene.order='logFC',

lfc.col=c('red','white','blue'))

#最后用画图搞一搞了

**ICGC数据库整理**

**（参考https://zhuanlan.zhihu.com/p/544873240）**

library(tidyverse)

library(data.table)

exp_seq1 <- read_delim("exp_seq.tsv.gz", "\t", escape_double = FALSE, trim_ws = TRUE)

exp_clinncal <- read.table("donor.tsv", header=T, sep="\t")

library(tidyverse)

**rpkm转tpm**

exp_seq1less <- exp_seq1[,c(1,5,8,9)]#取需要的列出来为一个新的文件

library(stringr)

> library(dplyr)

> library(tidyr)

> library(reshape2)

mat1 <- acast(exp_seq1less,gene_id~submitted_sample_id)#长宽转换

mat2=t(mat1)

write.csv(mat2,'0mat2.csv')

mat3<-read.csv('0mat2.csv')

library(data.table)

apply(mat1, 2, sum)

rpkmTOtpm <- function(mat1){

exp(log(mat1) - log(sum(mat1)) + log(1e6))

}

tpm2 <- apply(mat1, 2, rpkmTOtpm)

apply(tpm2, 2, sum)

**tpm转log2（tpm+1）**

library(dplyr)

tpm3<-log2(tpm2+1)

write.csv(tpm3,'0tpm3.csv')

tpm3<-read.csv('0tpm3.csv')

**提取目的基因**

exp_seq6 <- tpm3[tpm3$X == "ENSG00000141756", ]

exp_seq7 <- tpm3[tpm3$X == "ENSG00000164877", ]

exp_seq8 <- tpm3[tpm3$X == "ENSG00000196177", ]

write.csv(exp_seq6,'0exp_seq6.csv')

write.csv(exp_seq7,'0exp_seq7.csv')

write.csv(exp_seq8,'0exp_seq8.csv')

icgc_file<-read.csv('0exp_seq3in1.csv')

rownames(icgc_file) <- icgc_file[,1]

icgc_file=t(icgc_file)

write.csv(icgc_file,'0icgc_file.csv')

icgc_file<-read.csv('0icgc_file.csv')

icgc_file2<-read.csv('0exp_seq4lessless.csv')

rownames(icgc_file) <- icgc_file [,1]

rownames(icgc_file2) <- icgc_file2 [,2]

icgc_file_final <- left_join(icgc_file2, icgc_file, by="submitted_sample_id")

rownames(icgc_file_final) <- icgc_file_final [,1]

rownames(exp_clinncal) <- exp_clinncal [,1]

icgc_file_final_with_clinical <- left_join(icgc_file_final, exp_clinncal, by="icgc_donor_id")

icgc_file_final_with_clinical$OS.time <- icgc_file_final_with_clinical$donor_survival_time / 365

icgc_file_final_with_clinical$FKBP10p<- icgc_file_final_with_clinical$FKBP10*0.1995

icgc_file_final_with_clinical$MICALL2p<- icgc_file_final_with_clinical$MICALL2*0.3089

icgc_file_final_with_clinical$ACADSBp<- icgc_file_final_with_clinical$ACADSB*0.4429

icgc_file_final_with_clinical$Risk_Score<- icgc_file_final_with_clinical$FKBP10p+ icgc_file_final_with_clinical$MICALL2p- icgc_file_final_with_clinical$ACADSBp

rownames(icgc_file_final_with_clinical) <- icgc_file_final_with_clinical[,1]

icgc_file_final_with_clinical$Risk_Group <- ifelse(icgc_file_final_with_clinical$Risk_Score<median(icgc_file_final_with_clinical$Risk_Score),'Low Risk','High Risk')

icgc_finally <- icgc_file_final_with_clinical[order(icgc_file_final_with_clinical$Risk_Score,decreasing = F),]

icgc_finally$id <- c(1:length(icgc_finally$Risk_Score))

write.csv(icgc_finally,'0icgc_finally.csv',row.names = F)

icgc_finally0101<-read.csv('0icgc_finally0101.csv')

library(survminer)

library(survival)

fit <- survfit(Surv(OS.time, OS) ~ Risk_Group, data = icgc_finally0101)

fit

Call: survfit(formula = Surv(OS.time, OS) ~ Risk_Group, data = icgc_finally0101)

n events median 0.95LCL 0.95UCL

Risk_Group=High Risk 43 14 NA NA NA

Risk_Group=Low Risk 48 16 NA 5.27 NA

ggsurvplot(

fit,

data = icgc_finally0101,

censor = T,

censor.shape = "|", censor.size = 4,

conf.int = TRUE,

conf.int.style = "ribbon",

conf.int.alpha = 0.3,

pval = TRUE,

pval.size = 5,

legend = "top",

legend.title = 'Risk Score',

legend.labs = c("High Risk","Low Risk"),

xlab = "Years",

ylab = "Survival probablity",

palette = c('red','green'),

ggtheme = theme_bw(),

risk.table = TRUE,

risk.table.col = "black",

risk.table.title = 'Number at risk',

fontsize = 4,

risk.table.y.text = FALSE,

risk.table.height = 0.2,

)
